# Predicting diabetic cardiomyopathy in type 2 diabetes: development and validation of a nomogram based on clinical and echocardiographic parameters

**DOI:** 10.3389/fendo.2025.1641114

**Published:** 2025-08-08

**Authors:** Zilang Luo, Damao Pi, Tianlan Xi, Wenli Jiang, Feng Qiu, Jiadan Yang

**Affiliations:** ^1^ Department of Pharmacy, The First Affiliated Hospital of Chongqing Medical University, Chongqing, China; ^2^ College of Pharmacy, Chongqing Medical University, Chongqing, China

**Keywords:** diabetic cardiomyopathy, type 2 diabetes mellitus, nomogram, risk prediction model, echocardiographic

## Abstract

**Objective:**

Diabetic cardiomyopathy (DCM) is a myocardial dysfunction disorder driven by diabetes-associated metabolic disorders, significantly elevating the risk of heart failure in patients with type 2 diabetes mellitus (T2DM). We aimed to develop and validate a nomogram for individualized DCM risk prediction in T2DM populations.

**Methods:**

This retrospective study enrolled 525 consecutive T2DM patients admitted to our hospital (June 2022-June 2024). Participants were randomly allocated to training (70%) or validation (30%) cohorts. Baseline clinical characteristics, laboratory profiles, and echocardiographic parameters were collected. Predictors were identified via univariate then multivariate logistic regression, followed by nomogram construction. Model validation included: (1) internal validation via 1000 bootstrap resamples; (2) discrimination assessed by the area under the receiver operating characteristic curve (AUC-ROC); (3) calibration evaluated using calibration plots and the Hosmer-Lemeshow goodness-of-fit test; (4) clinical utility determined by decision curve analysis (DCA) and clinical impact curves (CIC).

**Results:**

Six independent predictors—age, duration of type 2 diabetes mellitus (T2DM Duration), systolic blood pressure (SBP), urinary albumin-to-creatinine ratio (UACR), left atrial diameter (LAD), and left ventricular posterior wall thickness at end-diastole (LVPWd)—were incorporated. The model showed excellent discrimination: AUC 0.947 (95% CI: 0.916-0.967) in training and 0.922 (95% CI: 0.870-0.956) in validation cohorts. Calibration indicated strong agreement (Hosmer-Lemeshow χ² = 9.2119, *P* = 0.3247). DCA and CIC confirmed clinical utility.

**Conclusions:**

This nomogram integrates routine clinical/echocardiographic parameters to predict DCM risk in T2DM patients, facilitating individualized risk stratification and guiding early cardioprotective interventions in high-risk populations.

**Clinical Trial Registration:**

https://www.chictr.org.cn/index.html, identifier ChiCTR2400093755.

## Introduction

Type 2 diabetes mellitus (T2DM) represents a global health crisis, with its prevalence and incidence exhibiting persistent upward trends. According to projections by the International Diabetes Federation (IDF), the global population with T2DM is anticipated to reach 700 million by 2045 (1). Beyond conventional micro- and macrovascular complications, T2DM directly contributes to myocardial pathology through diabetic cardiomyopathy (DCM)—a distinct entity characterized by diabetes-associated metabolic derangements that induce structural and functional cardiac alterations independent of coronary artery disease, valvular abnormalities, congenital heart defects, or other established etiologies of heart failure (HF) (2, 3). Epidemiological studies have demonstrated that cardiovascular mortality quadruples in diabetic populations compared to non-diabetic individuals (4), with DCM prevalence ranging from 10% to 21% among individuals with diabetes and associated mortality rates reaching 31% (5).

The pathogenesis of DCM is principally driven by metabolic dysregulation of carbohydrate and lipid homeostasis, triggering a cascade of pathophysiological events including oxidative stress generation, chronic inflammatory responses, endothelial dysfunction, and mitochondrial impairment (6, 7). These interconnected mechanisms collectively promote key pathological processes: cardiomyocyte hypertrophy, interstitial fibrosis, programmed cell death, coronary endothelial injury, and microvascular dysfunction (8, 9). Clinically, DCM manifests initially as elevated left ventricular filling pressures and diastolic dysfunction (10), progressing through stages of worsening diastolic impairment and eventual systolic failure, culminating in overt HF (11). Notably, asymptomatic left ventricular diastolic dysfunction frequently precedes symptomatic HF in T2DM patients, representing the earliest detectable functional manifestation of DCM (12). This insidious progression underscores the critical need for early detection and intervention to alleviate the escalating global burden of HF (13, 14).

Current diagnostic challenges persist due to the lack of disease-specific biomarkers, while advanced imaging modalities such as cardiac magnetic resonance imaging (CMR) remain cost-prohibitive for routine clinical implementation (15). Although prior studies have attempted to develop predictive models for DCM in T2DM patients, Existing tools exhibit certain limitations. First, certain models were derived from limited cohorts (e.g., one nomogram study with n = 84 cases), potentially compromising statistical power and increasing risks of overfitting or reduced generalizability (16). Second, some prediction frameworks rely exclusively on basic clinical characteristics (e.g., age, body mass index) and laboratory parameters (e.g., lipids, electrolytes), omitting cardiac structural/functional metrics essential for capturing DCM pathophysiology (17). To address these gaps, we developed and validated a clinical nomogram integrating routinely accessible clinical and echocardiographic parameters for DCM risk prediction in T2DM patients. This tool aims to enable early risk stratification, facilitating timely preventive strategies and targeted interventions to mitigate DCM-associated morbidity in high-risk populations.

## Methods

### Study design and participants

This study utilized electronic medical records from the Endocrinology Department of the First Affiliated Hospital of Chongqing Medical University (Chongqing, China). We consecutively screened patients admitted between June 2022 and June 2024, ultimately enrolling 525 eligible individuals with T2DM. Eligible participants were randomly allocated (7:3 ratio) to training (n = 367) and validation (n = 158) cohorts using computer-generated random numbers.

### Inclusion and exclusion criteria

The inclusion criteria included (1): Age ≥18 years (2); Diagnosis of T2DM is established based on the American Diabetes Association (ADA) criteria (18): Hemoglobin A1c (HbA1c) ≥6.5% or fasting plasma glucose ≥7.0 mmol/L or 2-Hour plasma glucose during oral glucose tolerance test (OGTT) ≥11.1 mmol/L or random plasma glucose ≥11.1 mmol/L; (3) Complete echocardiographic evaluation.

The exclusion criteria included: (1) Established cardiovascular pathology: (a) HF, established diagnosis or typical heart failure symptoms and signs (NYHA class II-IV symptoms) who were on long-term anti-HF medications; (b) hypertensive heart disease, documented history or uncontrolled severe hypertension; (c) coronary artery disease, known history of coronary artery disease, myocardial infarction, or coronary angiography demonstrating ≥50% luminal stenosis; (d) significant valvular disease, moderate-to-severe valvular stenosis/regurgitation confirmed by echocardiography; (e) congenital heart defects, clinically significant congenital heart disease verified by medical history or echocardiography. (2) Comorbid conditions: active malignancies, systemic infections (CRP >10 mg/L), hepatic insufficiency (Child-Pugh B/C), renal dysfunction (eGFR <30 mL/min/1.73m²); (3) Diabetes subtypes: Type 1 diabetes, gestational diabetes, secondary diabetes; (4) Incomplete core clinical parameters.

### Diagnosis of DCM

In accordance with the 2024 ESC Position Paper on DCM (endorsed by the Heart Failure Association and Working Group on Myocardial and Pericardial Diseases), DCM is defined as systolic and/or diastolic myocardial dysfunction occurring in patients with diabetes mellitus (19). Given this study’s focus on early-stage DCM risk prediction, we targeted the initial disease continuum: Diastolic dysfunction represents the earliest detectable phenotype of diabetic myocardial injury. Systolic dysfunction (LVEF <50%) typically indicates advanced disease incompatible with early warning objectives (2).

Therefore, through strict application of pre-specified inclusion/exclusion criteria, study participants were confirmed as DCM cases upon meeting the criteria for left ventricular diastolic dysfunction. Diastolic dysfunction was defined per the 2016 ASE/EACVI Recommendations for the Evaluation of Left Ventricular Diastolic Function, requiring ≥3 of the following echocardiographic criteria (20): (1) Septal e’ velocity <7 cm/s or Lateral e’ velocity <10 cm/s; (2) Peak tricuspid regurgitation velocity >2.8 m/s; (3) Early diastolic mitral inflow velocity to tissue Doppler mitral annular early diastolic velocity ratio (E/e’) >14; (4) Left atrial volume index (LAVI) >34 mL/m².

### Baseline data collection

Clinical characteristics and laboratory parameters were systematically collected through standardized protocols. Demographic and clinical variables included: age, sex, body mass index (BMI), duration of type 2 diabetes mellitus (T2DM Duration) (calculated from initial diagnosis to current hospitalization), heart rate, systolic blood pressure (SBP), diastolic blood pressure (DBP), smoking status, and alcohol consumption history. We obtained fasting venous blood samples were obtained within 24 hours of admission using standardized phlebotomy procedures. Complete blood counts - including white blood cells (WBC), neutrophils (NEU), lymphocytes (LYM), and platelets (PLT) - were analyzed using an automated hematology analyzer, with derived platelet-to-lymphocyte ratio (PLR) and neutrophil-to-lymphocyte ratio (NLR). Glycated hemoglobin (HbA1c) was measured by high-performance liquid chromatography, while biochemical profiles (triglycerides [TG], total cholesterol [TC], high-density lipoprotein cholesterol [HDL-C], low-density lipoprotein cholesterol [LDL-C], and serum creatinine [Scr]) were assessed using a clinical chemistry analyzer. First-morning midstream urine specimens were collected for urinary albumin-to-creatinine ratio (UACR) measurement via immunoturbidimetry. Estimated glomerular filtration rate (eGFR) was calculated applying the Chronic Kidney Disease Epidemiology Collaboration (CKD-EPI) equation (21). All data underwent dual-entry verification by independent investigators to ensure completeness and validity.

Standardized transthoracic echocardiography was performed by certified sonographers using a GE Vivid E95 ultrasound system (GE Healthcare) equipped with M5S transducers (1.5-4.6 MHz). Measured parameters included: left atrial diameter (LAD), left ventricular end-diastolic diameter (LVEDD), interventricular septal thickness at end-diastole (IVSd), left ventricular posterior wall thickness at end-diastole (LVPWd), relative wall thickness (RWT; 2×LVPWd/LVEDD), left ventricular ejection fraction (LVEF; Simpson’s biplane method). Left ventricular mass index (LVMI) was calculated using the Devereux formula (22). Measurement of diastolic function and tissue Doppler parameters: early diastolic mitral annular velocities (e’) were acquired using pulsed-wave tissue Doppler imaging (TDI) at the basal septal and lateral segments in the apical four-chamber view. Mitral inflow E-wave velocity was subsequently measured by placing the pulsed-wave Doppler sample volume at the mitral valve leaflet tips in the standard apical four-chamber view. The E/e’ ratiowas calculated as the ratio of mitral E-wave velocity to the average of septal and lateral e’ velocities. The LAVI was quantified in apical four- and two-chamber views using the biplane area-length method and indexed to body surface area. Additionally, peak TRV was obtained via continuous-wave (CW) Doppler in either the apical four-chamber view or dedicated right ventricular inflow view.

### Statistical analysis

All statistical analyses were conducted using R software (version 4.4.1; R Foundation) and IBM SPSS Statistics (version 25.0; IBM Corp). Continuous variables were assessed for normality through Shapiro-Wilk tests supplemented by histogram visualization. Normally distributed variables were expressed as mean ± standard deviation (SD) and compared using Student’s t-test, while non-normally distributed variables were reported as median (interquartile range [IQR]) with Mann-Whitney U test comparisons. Categorical variables were presented as frequencies (percentages) and analyzed by χ² test or Fisher’s exact test as appropriate. we performed univariate logistic regression analysis on the training cohort and the independent risk factors were further analyzed by multivariate logistic regression (Stepwise backward regression method). This model was visualized as a nomogram using the rms package in R with proportional scaling. Internal validation employed 1000 bootstrap resamples to estimate optimism-corrected performance metrics and mitigate overfitting risks. Model discrimination was evaluated via receiver operating characteristic (ROC) curve analysis reporting area under the curve (AUC) with 95% confidence intervals. Calibration was assessed using Hosmer-Lemeshow goodness-of-fit tests and calibration plots comparing predicted versus observed probabilities. Clinical utility was assessed through decision curve analysis (DCA) and clinical impact curves (CIC), with DCA quantifying the net benefit across threshold probabilities and CIC estimating true-positive versus false-positive classification rates. The P value of <0.05 indicated statistical significance.

## Results

### Baseline characteristics

**Figure 1:**
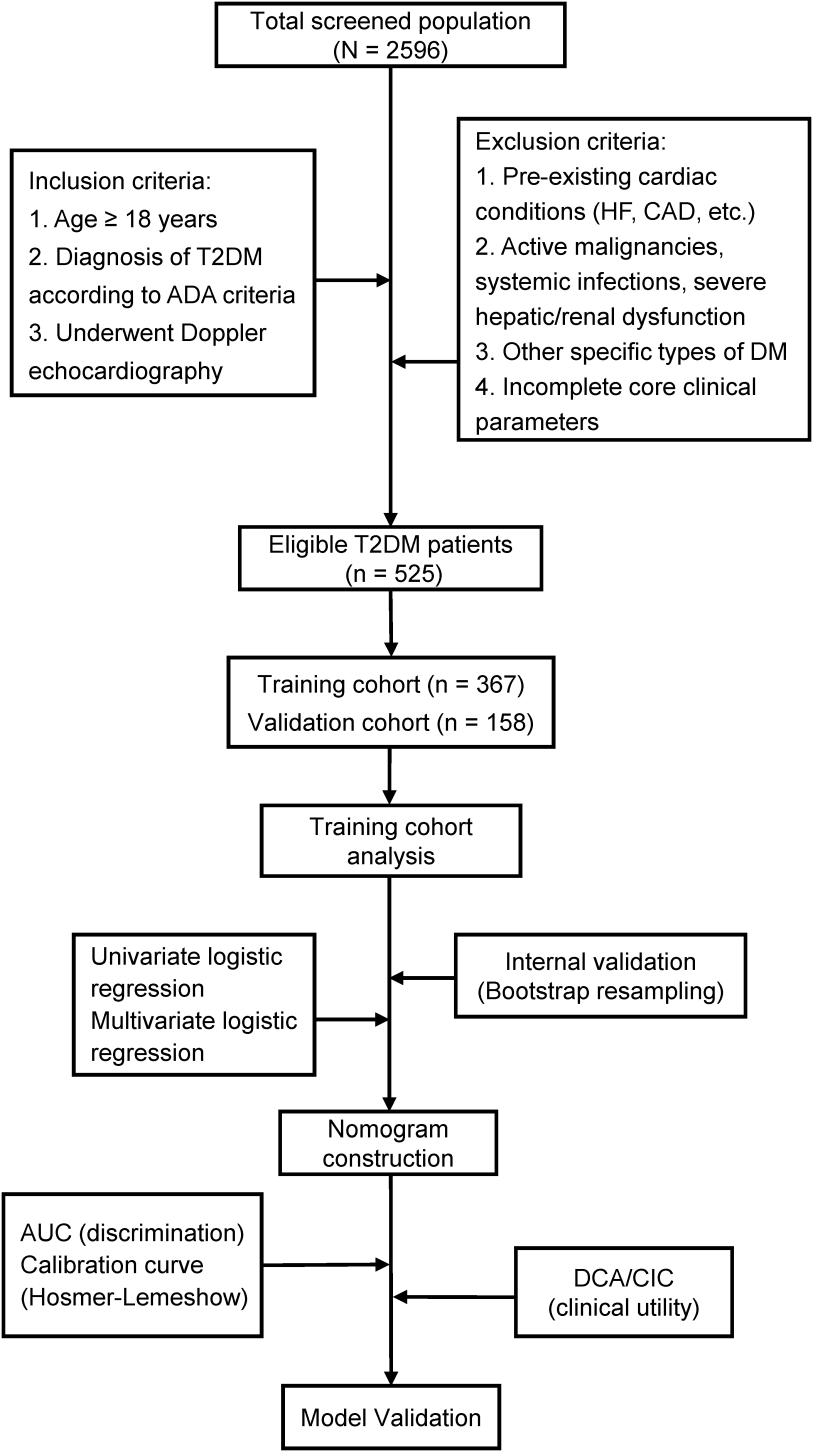
Flow diagram of study design.

The study flowchart is presented in **Figure 1**. The study cohort comprised 525 patients with T2DM, among whom 174 (33.1%) met diagnostic criteria for DCM. **Table 1** presents comparative analyses of clinical and echocardiographic parameters between the training (n = 367) and validation (n = 158) cohorts. The training cohort demonstrated a median age of 54.0 years (IQR 45.0-61.0) with male predominance (64.3%), while the validation cohort had comparable baseline characteristics: median age 54.0 years (IQR 45.0-63.0) and male proportion 67.1%. Intergroup comparisons revealed statistically significant differences only in smoking status (*P* = 0.047), with no clinically relevant disparities observed in T2DM duration, BMI, SBP, or other key variables. This baseline homogeneity supports the validity of the internal model validation procedures.

**Table 1:** The baseline characteristics of patients in the training and validation cohort.

Parameter	Training cohort (n = 367)	Validation cohort (n = 158)	χ²/z/t	*P*
Clinical information				
Age (years)	54.00 (45.00, 61.00)	54.00 (45.00, 63.00)	-0.388	0.698^a^
Gender (n, %)			0.377	0.539^c^
Female	131 (35.7)	52 (32.9)		
Male	236 (64.3)	106 (67.1)		
T2DM Duration (years)	8.00 (2.00, 14.00)	7.00 (2.00, 12.00)	-0.959	0.337^a^
BMI (kg/m^2^)	23.73 (21.85, 25.97)	24.33 (21.88, 26.77)	-1.233	0.218^a^
Heart rate (bpm)	88.00 (81.00, 96.00)	89.00 (80.00, 99.00)	-0.661	0.508^a^
SBP (mmHg)	129.00 (118.00, 144.00)	130.00 (119.00, 142.00)	-0.661	0.508^a^
DBP (mmHg)	79.22 ± 11.99	79.91 ± 11.30	-0.615	0.539^b^
Smoking (n, %)			3.932	0.047^c^
Yes	129 (35.1)	70 (44.3)		
No	238 (64.9)	88 (55.7)		
Drinking (n, %)			0.560	0.454^c^
Yes	111 (30.2)	53 (33.5)		
No	256 (69.8)	105 (66.5)		
WBC (10^9^/L)	6.53 (5.38, 7.78)	6.60 (5.26, 7.99)	-0.318	0.750^a^
PLR	129.32 (99.27, 157.95)	118.67 (94.02, 159.35)	-1.167	0.243^a^
NLR	2.39 (1.75, 3.29)	2.31 (1.74, 3.15)	-0.716	0.474^a^
SCr (μmol/L)	67.00 (55.00, 75.00)	67.00 (55.00, 78.25)	-0.129	0.898^a^
UACR (mg/g)	19.85 (11.30, 49.68)	17.00 (10.60, 47.23)	-0.400	0.689^a^
eGFR (mL/min/1.73m^2^)	105.20 (91.65, 117.60)	105.56 (93.22, 117.92)	-0.500	0.617^a^
TC (mmol/L)	4.39 (3.67, 5.21)	4.51 (3.83, 5.31)	-1.241	0.214^a^
TG (mmol/L)	1.57 (1.07, 2.69)	1.74 (1.11, 3.10)	-0.779	0.436^a^
HDL (mmol/L)	1.01 (0.83, 1.24)	1.02 (0.86, 1.24)	-0.440	0.660^a^
LDL (mmol/L)	2.62 (1.20, 3.21)	2.72 (2.07, 3.34)	-1.034	0.301^a^
HbA1c (%)	9.65 (7.58, 11.53)	9.60 (8.08, 11.53)	-0.568	0.570^a^
Echocardiographic parameters				
LAD (mm)	31.00 (29.00, 33.00)	31.00 (29.00, 32.00)	-0.644	0.519^a^
LVEDD (mm)	45.00 (42.00, 48.00)	45.00 (41.00, 48.00)	-1.618	0.106^a^
IVSd (mm)	10.00 (10.00, 11.00)	10.00 (10.00, 11.00)	-0.179	0.858^a^
LVPWd (mm)	10.00 (10.00, 11.00)	10.00 (10.00, 11.00)	-0.520	0.603^a^
RWT	0.43 (0.40, 0.45)	0.44 (0.41, 0.46)	-1.840	0.66^a^
LVMI (g/m²)	91.59 (81.18, 103.30)	86.92 (78.99, 102.83)	-1.756	0.079^a^
LVEF (%)	64.00 (61.00, 67.00)	63.50 (61.00, 66.00)	-0.464	0.643^a^

BMI, body mass index; DBP, diastolic blood pressure; eGFR, Estimated glomerular filtration rate; HbA1c, glycated hemoglobin; HDL-C, high-density lipoprotein cholesterol; IVSd, Interventricular septal thickness at end-diastole; LAD, Left atrial diameter; LDL-C, low-density lipoprotein cholesterol; LVEDD, Left ventricular end-diastolic diameter; LVEF, Left ventricular ejection fraction; LVMI, Left ventricular mass index; LVPWd, Left ventricular posterior wall thickness at end-diastole; NLR, neutrophil-to-lymphocyte ratio; PLR, platelet-to-lymphocyte ratio; RWT, relative wall thickness; SBP, systolic blood pressure; Scr, serum creatinine; T2DM Duration, duration of type 2 diabetes mellitus; TC, total cholesterol; TG, triglycerides; UACR, urinary albumin-to-creatinine ratio; WBC, white blood cells. ^a^Results shown as median and interquartile range and analyzed using Mann-Whitney U-test. ^b^Results shown as mean ± standard and analyzed using Student’s t-test. ^c^Chi-square test was used for proportions comparison.

### Univariate and multivariable logistic regression analysis

Univariate logistic regression analysis identified 17 variables significantly associated with DCM (*P <*0.05) in the training cohort. To mitigate overfitting and multicollinearity risks, we adhered to the events per variable (EPV) ≥10 principle (23), permitting inclusion of ≤12 variables given 125 DCM events. Variables were prioritized based on statistical significance (*P <*0.01), with 12 candidate variables retained for subsequent multivariate analysis. After performing multivariate logistic regression analysis (using backward stepwise regression), a variance inflation factor (VIF) less than 5 indicates acceptable multicollinearity, six independent predictors emerged (*P <0.05*): Age (OR = 1.046, 95% CI:1.008-1.088, *P* = 0.0198), T2DM Duration (OR = 1.254, 95% CI: 1.167-1.359, *P <*0.001), SBP (OR = 1.033, 95% CI:1.011-1.055, *P* = 0.0028), UACR (OR = 1.014, 95% CI:1.008-1.020, *P <*0.001), LAD (OR = 1.33, 95% CI:1.178-1.519, *P <*0.001), LVPWd (OR = 2.33, 95% CI:1.542-3.636, *P <*0.001). Detailed results are presented in **Tables 2** and **3**.

**Table 2:** Univariate logistic regression analysis of patients in the training cohort.

Parameter	OR (95% CI)	*P*
Clinical information		
Age (years)	1.087 (1.062-1.111)	< 0.001
Gender (n, %)	0.614 (0.393-0.958)	0.032
T2DM Duration (years)	1.244 (1.188-1.304)	< 0.001
BMI (kg/m^2^)	1.031 (0.973-1.093)	0.305
Heart rate (bpm)	0.991 (0.974-1.009)	0.33
SBP (mmHg)	1.035 (1.022-1.049)	< 0.001
DBP (mmHg)	0.982 (0.964-1.000)	0.046
Smoking (n, %)	0.766 (0.484-1.213)	0.255
Drinking (n, %)	1.133 (0.711-1.807)	0.599
WBC (10^9^/L)	1.029 (0.921-1.151)	0.613
PLR	1.005 (1.000-1.009)	0.031
NLR	1.539 (1.290-1.837)	< 0.001
SCr (μmol/L)	1.031 (1.020-1.042)	< 0.001
UACR (mg/g)	1.019 (1.013-1.025)	< 0.001
eGFR (mL/min/1.73m^2^)	0.956 (0.944-0.967)	< 0.001
TC (mmol/L)	0.880 (0.746-1.039)	0.132
TG (mmol/L)	0.956 (0.860-1.063)	0.406
HDL-C (mmol/L)	1.195 (0.604-2.366)	0.608
LDL-C (mmol/L)	0.740 (0.590-0.928)	0.009
HbA1c (%)	1.001 (0.924-1.086)	0.976
Echocardiographic parameters		
LAD (mm)	1.358 (1.248-1.477)	< 0.001
LVEDD (mm)	1.075 (1.017-1.135)	0.01
IVSd (mm)	3.144 (2.345-4.213)	< 0.001
LVPWd (mm)	3.214 (2.373-4.354)	< 0.001
RWT	1.820 (1.110-2.984)	0.018
LVMI (g/m²)	1.063 (1.047-1.081)	< 0.001
LVEF (%)	0.982 (0.931-1.037)	0.518

BMI, body mass index; DBP, diastolic blood pressure; eGFR, Estimated glomerular filtration rate; HbA1c, glycated hemoglobin; HDL-C, high-density lipoprotein cholesterol; IVSd, Interventricular septal thickness at end-diastole; LAD, Left atrial diameter; LDL-C, low-density lipoprotein cholesterol; LVEDD, Left ventricular end-diastolic diameter; LVEF, Left ventricular ejection fraction; LVMI, Left ventricular mass index; LVPWd, Left ventricular posterior wall thickness at end-diastole; NLR, neutrophil-to-lymphocyte ratio; PLR, platelet-to-lymphocyte ratio; RWT, relative wall thickness; SBP, systolic blood pressure; Scr, serum creatinine; T2DM Duration, duration of type 2 diabetes mellitus; TC, total cholesterol; TG, triglycerides; UACR, urinary albumin-to-creatinine ratio; WBC, white blood cells.

**Table 3:** Multivariate logistic regression analysis of patients in the training cohort.

Variable	β	SE	Wald	OR (95% CI)	*P*
Intercept	-27.798	3.713	56.046	–	< 0.001
Age	0.045	0.019	5.425	1.046 (1.008-1.088)	0.0198
T2DM Duration	0.226	0.039	34.006	1.254 (1.167-1.359)	< 0.001
SBP	0.032	0.011	8.889	1.033 (1.011-1.055)	0.0028
NLR	0.010	0.167	0.003	1.010 (0.728-1.400)	0.954
Scr	-0.001	0.016	0.006	0.999 (0.968-1.030)	0.937
UACR	0.014	0.003	21.192	1.014 (1.008-1.02)	< 0.001
eGFR	-0.009	0.011	0.721	0.991 (0.969-1.012)	0.396
LDL-C	-0.032	0.194	0.027	0.968 (0.662-1.417)	0.868
LAD	0.285	0.065	19.452	1.33 (1.178-1.519)	< 0.001
IVSd	0.189	0.366	0.268	1.208 (0.590-2.474)	0.605
LVPWd	0.846	0.218	15.074	2.33 (1.542-3.636)	< 0.001
LVMI	0.016	0.013	1.705	1.017 (0.992-1.042)	0.192

eGFR, Estimated glomerular filtration rate; IVSd, Interventricular septal thickness at end-diastole; LAD, Left atrial diameter; LDL-C, low-density lipoprotein cholesterol; LVPWd, Left ventricular posterior wall thickness at end-diastole; LVMI, Left ventricular mass index; NLR, neutrophil-to-lymphocyte ratio; SBP, systolic blood pressure; Scr, serum creatinine; T2DM Duration, duration of type 2 diabetes mellitus; UACR, urinary albumin-to-creatinine ratio.

### Development of the nomogram prediction model

**Figure 2:**
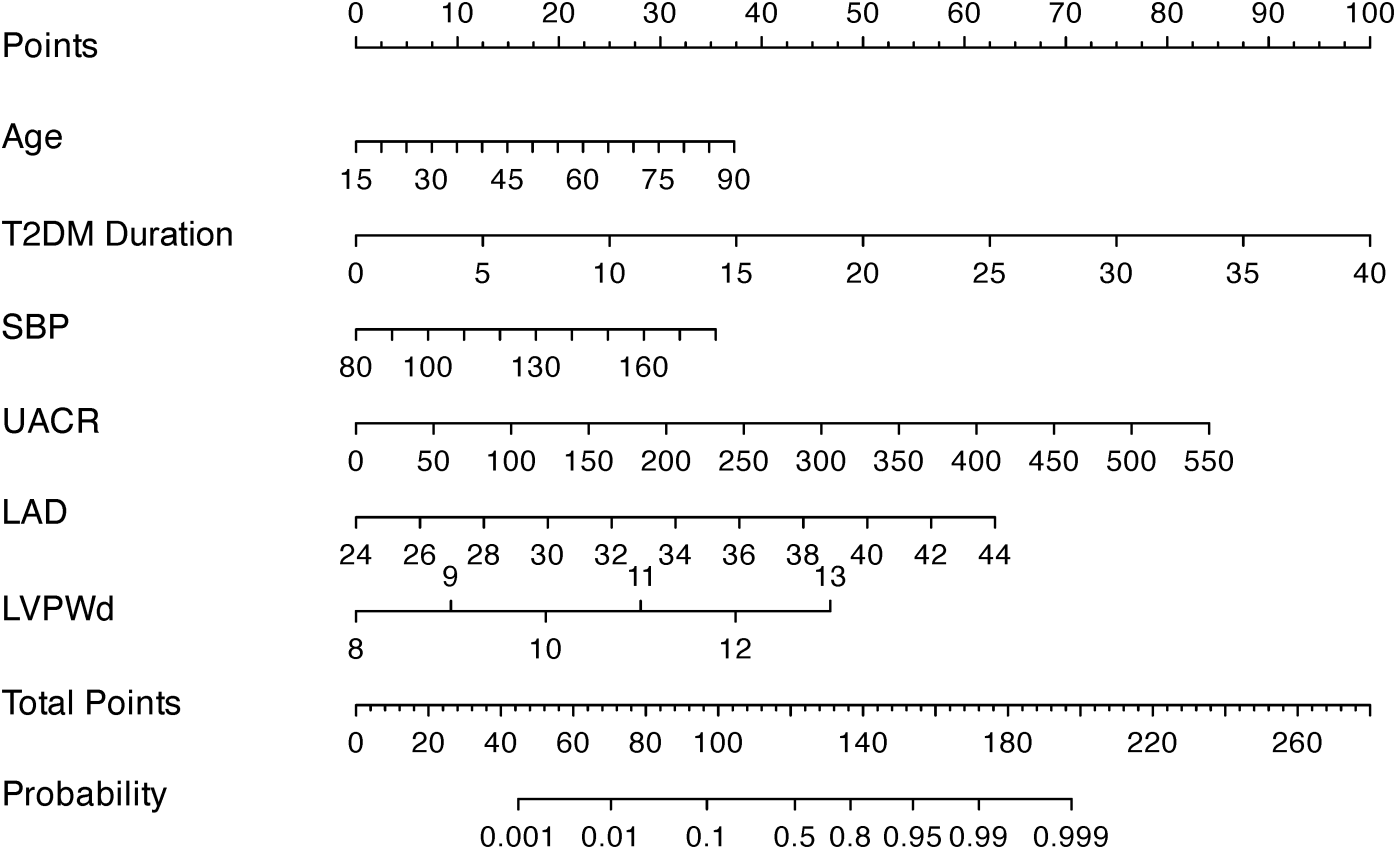
A nomogram integrating clinical indicators (age, T2DM Duration, SBP, UACR) and echocardiographic parameters (LAD, LVPWd) to predict DCM risk in T2DM patients.

The nomogram (**Figure 2**) was constructed to visualize the final multivariable logistic regression model. Point values assigned to each predictor were automatically calculated based on their corresponding regression coefficients (β-coefficients) using the rms package in R software. This mathematical transformation process ensures that the point scale accurately preserves both the relative weights of variables within the model and the risk differences between categorical levels of individual predictors.

**Figure 3:**
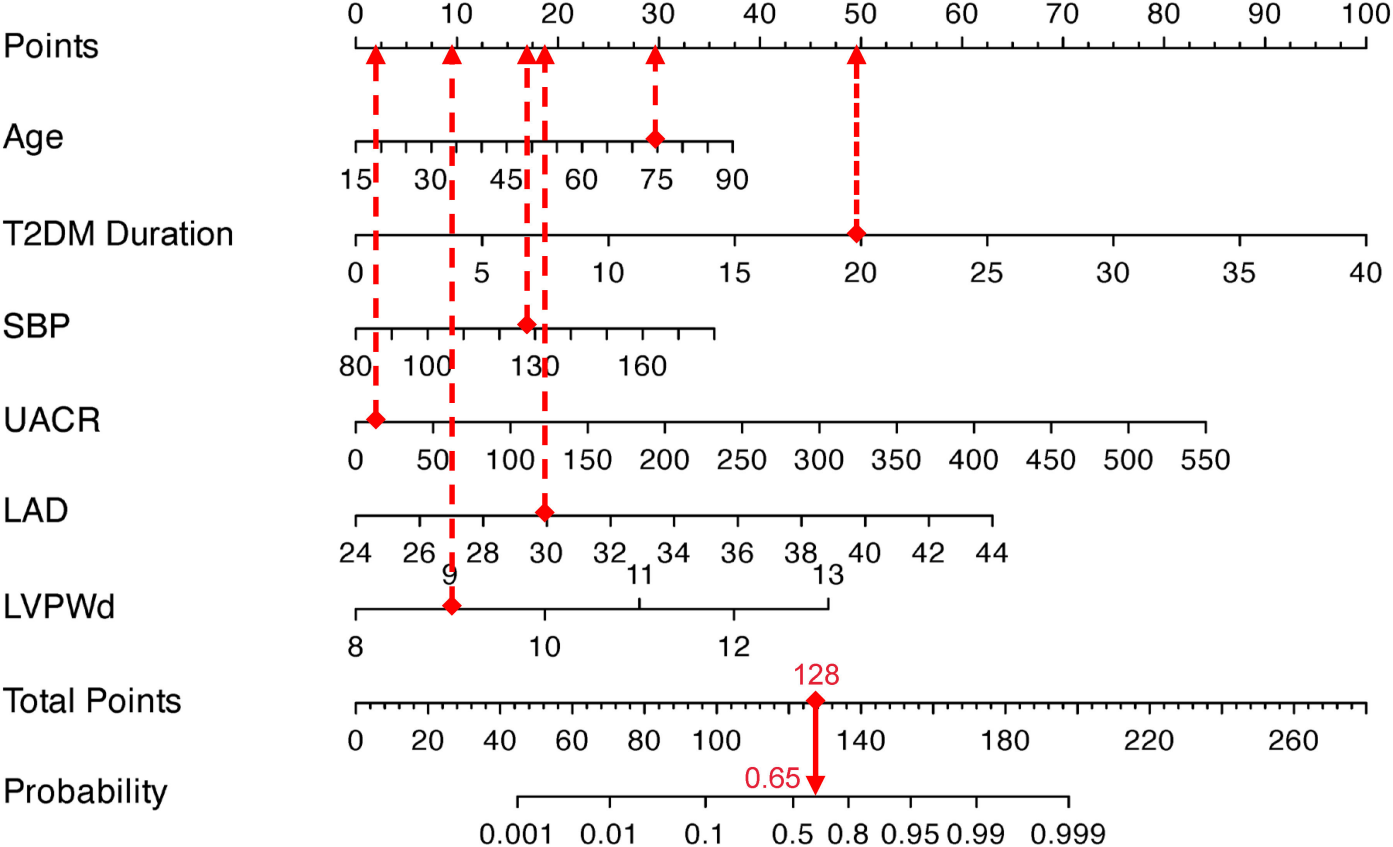
Visualization application of the nomogram prediction for the risk of DCM in patients with T2DM.

To apply the model, clinicians first assign individualized scores by mapping a patient’s clinical parameters to the corresponding variable axes. These scores are then summed to calculate a total risk score, which is subsequently projected onto the probability axis to estimate the personalized likelihood of DCM. For instance, a 75-year-old patient with T2DM, a 20-year disease duration, SBP of 129 mmHg, UACR of 13.6 mg/g, LAD of 30 mm, and LVPWd of 9 mm would accumulate a total score of 127.5 points, corresponding to a 65% predicted probability of DCM (**Figure 3**). This integrative tool enables rapid risk stratification and supports clinical decision-making for early intervention in high-risk populations.

### Model performance

**Figure 4:**
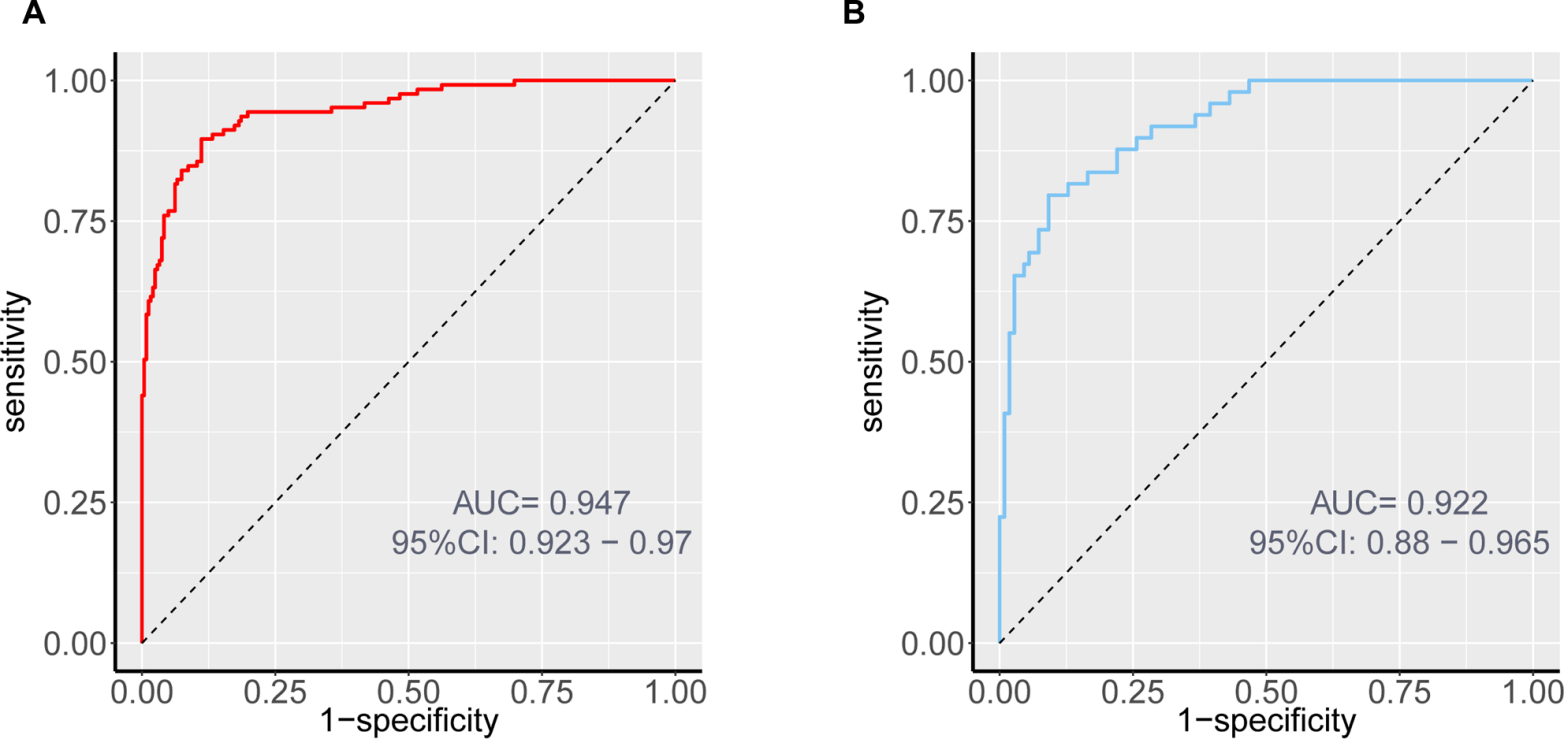
ROC curves of the nomogram prediction for the risk of DCM in patients with T2DM in the training cohort **(A)** and validation cohort **(B)**.

**Figure 5:**
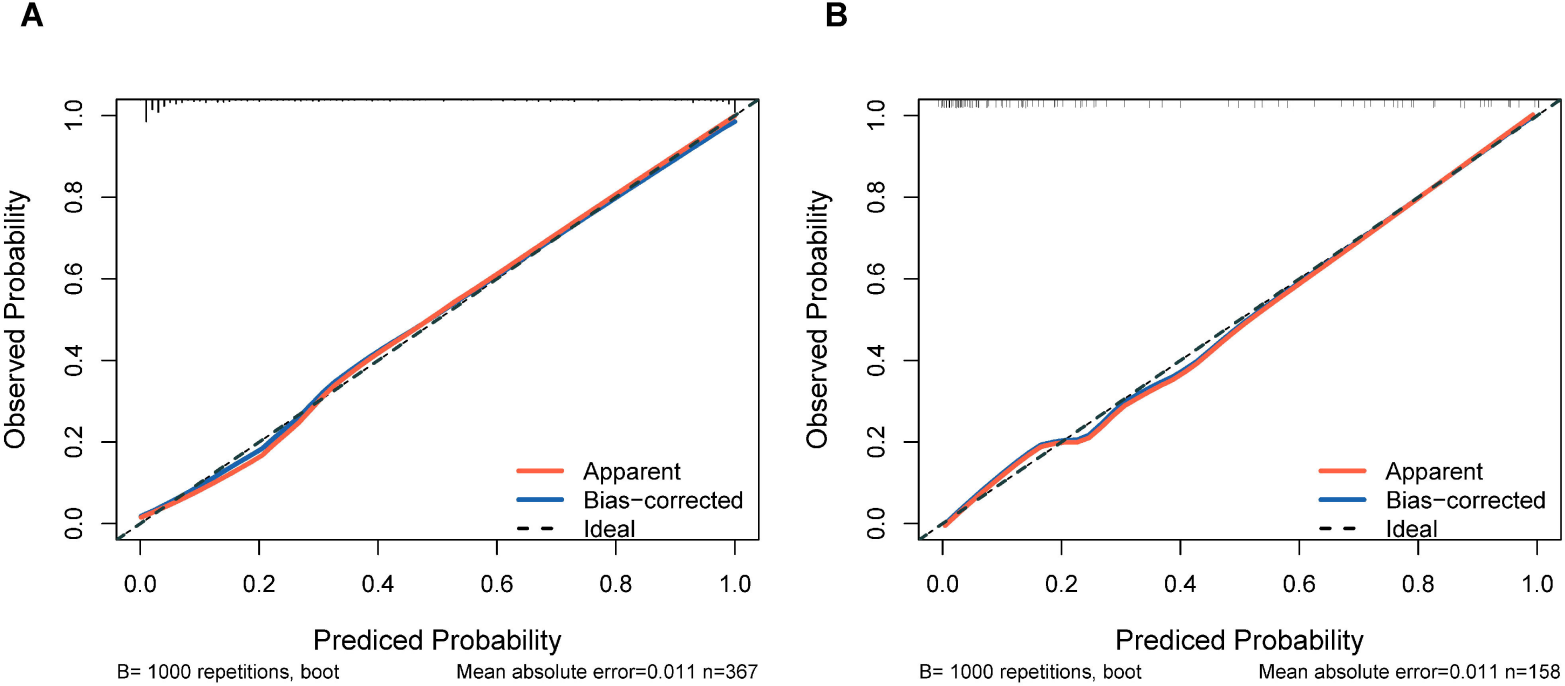
Calibration plots of the nomogram prediction for the risk of DCM in patients with T2DM in the training cohort **(A)** and validation cohort **(B)**.

Internal validation through 1000 bootstrap resamples demonstrated robust model discrimination, with a C-index of 0.9376 (95% CI: 0.9024-0.9728), indicating minimal overfitting risk. ROC analysis revealed excellent predictive accuracy in both training (AUC = 0.947, 95% CI: 0.916-0.967) and validation cohorts (AUC = 0.922, 95% CI: 0.870-0.956), surpassing the clinical utility threshold (AUC >0.75) (**Figure 4**). Calibration plots exhibited strong concordance between predicted probabilities and observed outcomes across risk strata (**Figure 5**). The Hosmer-Lemeshow test confirmed adequate model calibration (χ² = 9.2119, *P* = 0.3247), with non-significant deviation from ideal prediction (*P >*0.05).

### Model clinical utility

**Figure 6:**
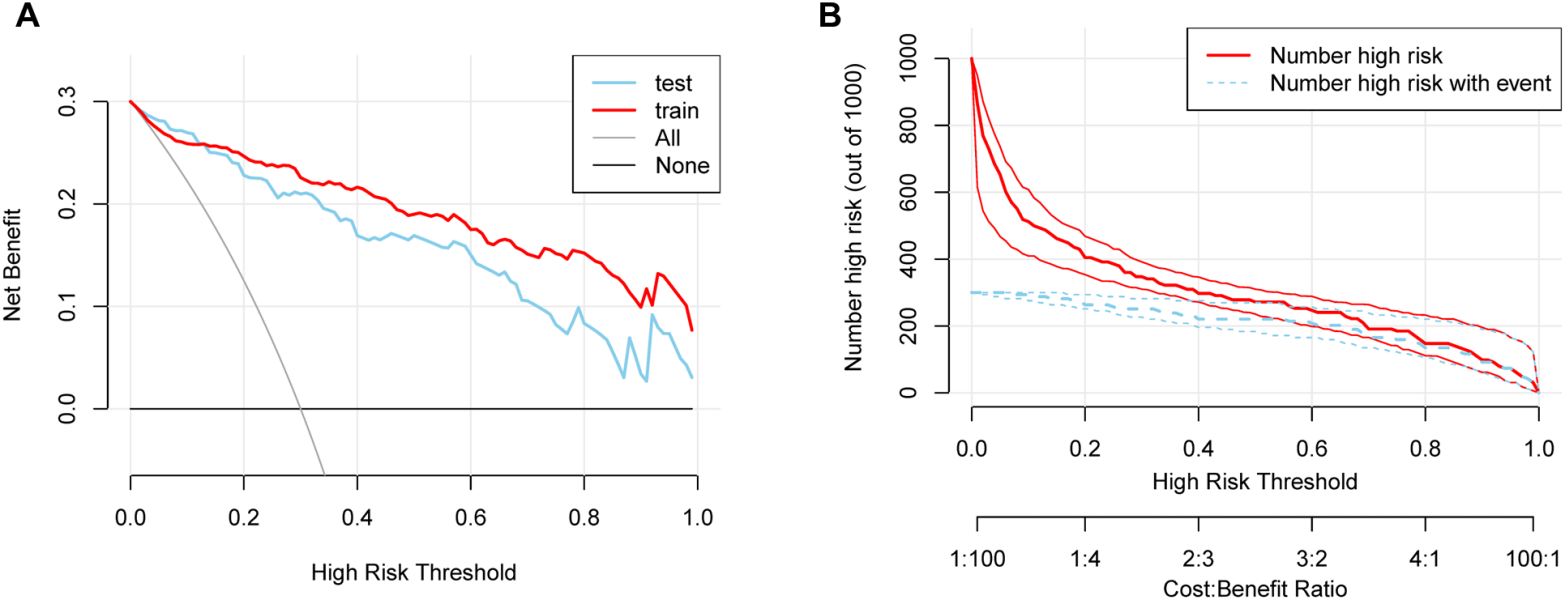
**(A)** DCA demonstrating the clinical utility of the nomogram across various risk thresholds. **(B)** CIC showing the relationship between predicted high-risk cases and actual event occurrences.

The DCA of the nomogram demonstrated superior net clinical benefits compared to default strategies (“Treat All” or “Treat None”) across low-to-medium risk thresholds (0.0-0.8), with both training and validation cohort curves exhibiting parallel trajectories and minimal divergence (**Figure 6A**). This consistency indicates robust generalizability without significant overfitting. CIC analysis further revealed high concordance between model-predicted high-risk cases and actual event occurrences at thresholds >0.6, highlighting its precision for resource-intensive interventions (**Figure 6B**). Integrating cost-benefit ratios, we recommend adopting flexible threshold selection (0.4-0.8) to optimize risk stratification and healthcare resource allocation based on clinical priorities and resource availability.

## Discussion

### Summary and interpretation of key findings

HF secondary to DCM represents a major contributor to premature mortality in patients with T2DM. While prior studies have established associations between echocardiographic parameters, biomarkers, and HF risk in diabetes (14, 24, 25), limited research has focused on predicting DCM progression in T2DM populations. To address this gap, we conducted a retrospective analysis of hospital-based data, identifying conventional clinical indicators (age, T2DM Duration, SBP, UACR) and cardiac structural remodeling markers (LAD, LVPWd) as independent predictors of DCM. Leveraging these factors, we developed and validated a clinically practical nomogram model. These results advance early DCM risk stratification and provide a framework for targeted clinical interventions.

The progression of DCM in patients with T2DM is synergistically driven by aging and prolonged hyperglycemic exposure. Extensive evidence identifies age as a critical determinant of cardiac dysfunction in T2DM populations (26–28), primarily through age-related cardiovascular remodeling characterized by myocardial stiffening, atherosclerotic changes, and metabolic dysregulation. These alterations establish a pathological substrate that amplifies diabetes-induced cardiac injury, particularly in elderly individuals with compromised metabolic homeostasis (26). Furthermore, diabetes duration emerges as an independent predictor of cardiovascular morbidity (29–31), reflecting cumulative hyperglycemic damage mediated by chronic oxidative stress, endothelial dysfunction, and cardiomyocyte apoptosis (29). The interplay between aging and diabetic metabolic disturbances creates a vicious cycle: Age-dependent myocardial fibrosis and diastolic impairment are exacerbated by persistent hyperglycemia, which accelerates glycation end-product accumulation and reactive oxygen species generation (14). This synergy explains why elderly patients with extended T2DM duration exhibit more severe myocardial injury and earlier onset of diastolic dysfunction (32). Our findings highlight the clinical imperative to incorporate both aging and glycemic exposure duration into DCM risk stratification frameworks, enabling targeted monitoring of high-risk subgroups.

Elevated blood pressure is a well-established risk factor for T2DM (33, 34), with diabetic populations exhibiting higher average blood pressure levels. A cross-sectional study in high-altitude Chinese populations identified hypertension and hyperglycemia as critical contributors to increased LVDD risk (35). Further supporting this, a 2018 clinical comparative study demonstrated a direct association between SBP and diastolic dysfunction severity (36). While diabetes alone rarely causes isolated myocardial impairment, its synergistic interactions with hypertension, obesity, and chronic kidney disease often amplify cardiac damage (19). In our study, elevated SBP emerged as a key driver of DCM progression. Mechanistically, prolonged hypertension induces myocardial hypertrophy and fibrosis, exacerbating cardiac workload and thereby aggravating LVDD in T2DM patients.

The UACR serves as a sensitive indicator of diabetic nephropathy, with renal impairment demonstrating strong cardiometabolic associations (37). Prevalent LVDD has been documented across stages of moderately increased (microalbuminuria) and severely increased albuminuria (macroalbuminuria), whereas left ventricular systolic dysfunction predominantly manifests in subgroups with advanced macroalbuminuria (38). Seminal investigations, including *The Strong Heart Study*, established associations between albuminuria, left ventricular mass index, and diastolic impairment. Notably, albuminuria independently correlates with multisystem damage irrespective of diabetes status, elevating risks of atherosclerotic cardiovascular events (myocardial infarction, ischemic stroke, cardiac mortality) and congestive heart failure (39). This aligns with our findings, reinforcing UACR’s dual role as both an early diabetic nephropathy marker and an independent predictor of cardiovascular outcomes, underscoring the cardiorenal continuum in diabetes pathophysiology.

In this study, LAD and LVPWd were identified as predictive indices, highlighting the critical role of cardiac structural remodeling in early DCM diagnosis. Left atrial enlargement and increased LVPWd in diabetic populations reflect adaptive cardiac responses to chronic hemodynamic stress, particularly in hypertensive conditions, ultimately progressing to myocardial dysfunction and heart failure (10, 40). A pooled analysis of three U.S. cohort studies (n = 10,208 participants without baseline cardiovascular disease or heart failure) demonstrated significantly higher prevalence of echocardiographic structural abnormalities—including left atrial dilation and left ventricular hypertrophy—among patients with T2DM. These abnormalities independently predicted 5-year incident heart failure risk (41, 42). Machine learning-based models for early DCM risk stratification similarly prioritize left ventricular mass and chamber size as key predictors (43). Our findings reinforce the necessity of proactive echocardiographic screening in T2DM patients with suboptimal glycemic control to detect structural cardiac alterations, enabling timely intervention to mitigate adverse outcomes.

This study revealed a noteworthy phenomenon: HbA1c—traditionally recognized as a key metabolic control indicator for diabetic complications—failed to demonstrate independent predictive value in our model. We postulate three underlying mechanisms:

(1) Metabolic memory effect (44): Persistent myocardial alterations (e.g., fibrosis and advanced glycation end-product deposition) induced by chronic hyperglycemia endure beyond glycemic correction. Consequently, baseline HbA1c inadequately reflects cumulative damage, explaining the superior predictive capacity of T2DM Duration. (2) Hierarchical masking in modeling: As disease progresses to structural remodeling stages (e.g., left ventricular hypertrophy/left atrial enlargement), imaging biomarkers may supersede upstream metabolic indicators. Concurrently, the synergistic modulation of glycemic exposure by age and diabetes duration further diminishes HbA1c’s contribution. (3) Temporal limitations of measurement: HbA1c captures only short-term (2-3-month) glycemic averages, failing to account for long-term cumulative myocardial injury from glycemic variability—a critical factor in chronic progressive disorders like DCM (45).

### Clinical significance and practical applications

Given the elevated risk of HF in patients with T2DM, early detection and intervention of DCM are critical for HF prevention. Predictive models enable clinicians to stratify high-risk individuals during the asymptomatic phase, facilitating timely implementation of tailored interventions—such as lifestyle modifications, metabolic parameter optimization, and evidence-based pharmacotherapy—to reduce cardiovascular morbidity (46). Current preventive strategies for T2DM-associated cardiovascular disease include: 1. Lifestyle intensification: Structured dietary control, weight management, and physical activity regimens (47). The European Society of Preventive Cardiology guidelines advocate for individualized exercise regimens tailored to patient-specific characteristics to optimize cardiovascular adaptation and metabolic homeostasis (48); 2. Metabolic control: Tight regulation of glycemic, blood pressure, and lipid profiles; 3. Cardioprotective pharmacotherapy: Prioritization of glucose-lowering agents with proven cardiovascular benefits, notably sodium-glucose cotransporter 2 inhibitors (SGLT2i). Critically, the therapeutic value of SGLT2i extends beyond glycemic control and cardiovascular protection. Their efficacy aligns profoundly with the concept of the cardiorenal metabolic syndrome (CRMS), which recognizes the intricate interplay and share pathophysiological mechanisms linking T2DM, cardiovascular diseases, and chronic kidney disease (49). A multinational cohort study (N = 309,056) demonstrated that SGLT2i initiation significantly reduced HF hospitalization risk and all-cause mortality compared to alternative therapies (50). Importantly, landmark trials have consistently shown that SGLT2i also provide significant renoprotection, reducing the risk of worsening kidney function, end-stage kidney disease, and death from renal causes (51). This dual cardiorenal benefit uniquely positions SGLT2i as foundational therapy for patients with T2DM and DCM, who frequently exhibit features of CRMS.

The early initiation of SGLT2i in high-risk populations, particularly those identified with early-stage DCM within the spectrum of CRMS, remains a critical priority. Despite robust evidence supporting their cardioprotective efficacy, widespread clinical adoption of SGLT2i is hindered by cost-related barriers and limited accessibility in resource-constrained settings (52). To reconcile this disparity, risk stratification strategies leveraging predictive models offer a pragmatic solution: targeted prioritization of SGLT2i therapy for individuals identified with early-stage DCM and/or concomitant renal risk markers through validated risk algorithms. Furthermore, integrating predictive models into personalized medicine frameworks holds transformative potential. By synthesizing patient-specific clinical indicators (including those reflecting cardiorenal metabolic risk, such as albuminuria, eGFR trajectory, along with cardiac structural/functional parameters), these tools enable clinicians to formulate precision-guided management plans tailored to individual risk trajectories. This approach not only enhances therapeutic efficacy by addressing the multifaceted nature of CRMS but also optimizes healthcare resource utilization, ultimately improving patient-reported outcomes and quality of life.

### Study limitations and future directions

Despite the insights provided, this study has several limitations. First, the single-center retrospective design resulted in a relatively limited sample size, potentially restricting the generalizability of the findings. Second, retrospective data collection introduces challenges in fully accounting for unmeasured confounders. To address these limitations, future research should validate the model’s validity and reliability through large-scale, multicenter prospective studies. Additionally, investigations into novel biomarkers and advanced imaging parameters are warranted to enhance predictive precision and clinical utility.

## Conclusion

We developed a validated nomogram integrating clinical indicators (age, T2DM Duration, SBP, UACR) and echocardiographic parameters (LAD, LVPWd) to predict DCM risk in T2DM patients. This tool enables early risk stratification, supporting timely interventions to reduce heart failure progression. Further multicenter validation and integration of novel biomarkers are warranted to enhance clinical utility and address global diabetes-related cardiovascular burdens.

## Data Availability

The raw data supporting the conclusions of this article will be made available by the authors, without undue reservation.
